# Amalgam

**Published:** 1863-06

**Authors:** 


AMALGAM.—It is not to try to prove what is *right* or what is *wrong* that we venture to speak again on the subject of amalgam; it is the business and duty of every one engaged in our noble art to help each other to arrive at the truth as fast as possible, and perhaps we would never have referred to this subject again but for a paper which appeared in the May number of the *Dental Cosmos*, by Thomas Burgh. In it he says, "the novice in the dental profession finds it extensively employed in filling teeth, but which is the subject of such varied comment as to bewilder him as to whether it is just or expedient to employ it in practice." The subject has been discussed for twenty five years at least, and we should suppose that ere this the novice would find no difficulty in deciding what course to pursue. An operator may pursue the quiet tenor of his way, and do many things which are not strictly within the bounds of professional propriety, and not fall under the law of censure; but when he openly advocates such things, it is quite different. The best of men have fallen in such endeavors. We have always objected to amalgam, and wrote against it twenty years ago, but never complained that dentists used it as an expedient, in some cases. The writer says, "let us examine the formal objections urged against its use: first, that it is deleterious to the general health; and second, that it is ineffectual in preserving the teeth. The former is the more serious of the two, and is supposed to originate in medical knowledge." We never urged any objections to it as merely "formal;" they were urged in good faith, and under convictions that we had not only *practical* but *medical* evidence that what we reported was correct and reliable. We but recently gave some cases in the *Dental Cosmos*, which were well authenticated, as well from our own observation as those furnished by medical men of varacity and ability. He further says, "let us survey this objection from the stand-point of reason and analogy." And further, "I have never heard this charge from that part of the world where, from the attention bestowed on medicine, it might be supposed to be best understood." As far as opinion goes, for our part, we do not believe that the medical profession is any better qualified to decide upon such matters in any part of the world than in this country. It is a dangerous charge, but one which the medical profession can, doubtless, defend better than we can. We are not willing to admit that there is any "reason" in the comparison, or "analogy." We believe the converse is true when we reflect upon the career the Craucours met with when first introduced into this country. The writer further adds, "if any physician, whom we know to be honest, skillful, and truthful, should, after a full investigation of the effects of amalgam upon the system, pronounce it injurious, any fair-minded man should accept even his mere opinion as entitled to weight in the matter." The old American Society held among its members some of the best-informed medical men in the country, and the ablest and most experienced practitioners, and they pronounced against it, and expelled members who would not sign a pledge not to use it in practice. We obtained our first impression against it twenty-five years ago, this summer, from one of the first surgeons in the City of Philadelphia, the late Dr. Geo. McClellan, while attending a case of surgery with him. He was laboring under, what he believed to be, ptyalism from amalgam plugs in his mouth; he enjoined upon us never to employ it, and we have kept the pledge. He was a man of extremely nervous and impressible temperament, especially to mercury. It is these idiosyncrasies which make medical men object to it, but which does not seem to have engaged the attention of the writer we refer to. We do not know a medical man in the whole round of our acquaintance who is favorable to the use of amalgam. This alone would forever prevent us from using it, but we are as much disposed to excuse a young man's shortcomings as any one in the profession; it is for him we have labored our life long. We know the difficulties which beset his way—we were young once too; but every plug of amalgam he puts in retards the rise of his reputation, and every one the older practitioner puts in is only tolerated because he can afford to do it, and if he uses it indiscriminately, and advocates it, he is bound to fall; every man will take the level of that which he professes to do. It will not be out of place here to add that the celebrated Dr. T. W. Evans, of Paris, some years since modified the formula of amalgam by adding cadmium, [see his renouncement,*] and which he believed would get rid of one, at least, of the objections to its discoloration. He sent to us the names of many of the first European dentists to prove to us that it was a superior article, (the history of it is known, and we need not dwell upon it.) We wrote to him disapproving of it; he returned for his answer that "it was the first time in his life he had departed from the wholesome advice given by us, and he now felt its importance more than ever;" then followed his recantation in all the journals of the day. Next came the late Dr. E. Townsend. After having been among the most prominent opponents of amalgam, he thought he had discovered a modification of it, and became one of its most powerful advocates,† and the large reputation which he enjoyed induced a wholesale use of it. He followed with a series of articles advocating its use; we, on the other hand, wrote several articles against it, which ended in causing him to renounce it in the most unqualified terms.* There will be no "bewilderment" if any one in starting out will study the history of the subject, especially as connected with these celebrated operators, and whose opinions are entitled to some weight. How much we have had to do with influencing this latter eminent man may be explained by the publication of the two following letters, and we only regret that an earlier opportunity has not been afforded us to show the nobleness of his spirit, and the honesty of his purpose for the profession's good. If the careful review of his recantation, and the discussion which led to it, has not done

---

*See Dental News Letter, vol. iii. No. 1, page 9, and vol. iii. No. 2, page 44.

†Ibid., April, 1851.

*See Dental News Letter, vol. xi. No. 3, p. 169.

sufficient to elucidate the subject of amalgam, where are we to look for more light? J. D. W.

Dear Doctor:—I have received your kind note, and thank you for the credit you do my honesty of purpose. On Saturday, during the pauses of suffering from disease, I wrote what I now send you for the *News Letter.* I hope it is not *too late*, and also that it will meet your views. Will you do me the favor to send it, and not let it fail to appear?

March 2, 1853.

My Dear Sir:—I sent back the amalgam fillings to you without comment, as I was then too feeble, from an attack of influenza, to write.

I will now say to you that I am very sorry I ever was induced to advocate it, as I see it has been grossly misused, and as it has also failed *me* in the very cases where I most relied upon it. I have thrown it aside entirely, emptied my bottles, and *will never* use it again in *any* case. My brother C., my brother Edward, and my brother-in-law, Mr. Kirk, have also pledged themselves to me never to employ it.

I shall appear in the next number of the *News Letter*, when I hope to make my recantation clear to all men. I very much regret it, and shall not be ashamed to own up.

In one thing, in the past year, my conscience is clear to my patients, and to the profession—I have taken unusual time, pains, and care with my gold fillings, and, not having been so crowded as to be uncomfortable, I think I may say have put in more perfect "stoppings" than ever I did. This little brag I hope you will think pardonable as an offset to some of my *black* deeds; and, besides, *you* know I can fill teeth. Very respectfully yours,

Dr. J. D. White. E. Townsend.

[*Dental Cosmos.*

Poisoning from Chloroform.—An unknown quantity of chloroform was swallowed as a remedy for sleeplessness, and was effectual. The patient was for several hours insensible, as though the anæsthetic had been inhaled. He then recovered his sensibility, but died under the violent re-action which ensued.—*Medical Times and Gazette.*

Deaths from Chloroform.—In the month of January last, some editorial comments were suggested to us by the mention in the *Medical Times and Gazette* of November 22, 1862, of the account of two deaths, which had then recently been caused, in one London hospital, by the inhalation of chloroform. Our attention is again attracted to the subject by the statement of a correspondent of the same journal for April 11, 1863, that three more deaths have happened in the London hospitals, and that these, like the previous ones, have passed unnoticed by the medical journals. In a second communication to the *Medical Times and Gazette* of April 25th, this writer himself narrates them in the following language. "A few words will prove useful as to the latest deaths from chloroform in London. One is noted last week. A young woman, aged 29, Selina L., Edgeware Road, who died in one instant, apparently of syncope, as she was about to have a small tumor of the gum (epulis?) removed. No one to blame, it being one of those singular and miserable cases of idiosyncrasy that can scarcely be anticipated. A second case of death was at Guy's Hospital. A man put under chloroform for a dislocation to be reduced. He bore the anæsthetic one day well, but the reduction was not effected, and he was desired to come again, I think, the next day; but he was only half way under chloroform when his death, as a flash of lightning, suddenly occurred. The third case was removal of a small tumor, I believe an asphyxia case. This and the fourth, somewhat doubtful, are described in the *Social Science Review.*"

Such are the flippant terms in which the loss of four lives is referred to, and that, too, when it is wholly within the power of man to avert these constantly recurring catastrophes. Nor are these all the "recent deaths" from this anæsthetic, for in the *Lancet* of May 16, 1863, an inquest is stated to have been held "on Monday last" over the body of a Dr. Evans, of Dalston, near Carlisle, who also died of chloroform, which it appears he was in the habit of using for the relief of pain. If we are to believe the astounding statement contained in an elaborate article on the use of chloroform in obstetrics, to be found in the May number of the *Dublin Quarterly* (p. 362), that one individual claims, as his own personal experience, "to have seen about five hundred cases restored to life, or rescued, after they were pronounced dead" from the effects of this agent, is it not surprising that fatal results should not occur with even greater frequency than they do? We gather these items from the single batch of English journals which the last steamer has just brought us. Yet with all their significance, and admitting, as they seem ready to do, that chloroform is on its trial, its advocates are willing to amuse themselves with the idea that "a Faradisation circuit, with an interrupted, broken current," applied by sticking pins into the phrenic nerve and the diaphragm, is the long-looked for antidote to its deadly influence, capable of restoring life in any one of the above cases, had its aid been properly invoked.

Really, the Royal Medico-Chirurgical Society appear to have but a limited choice left open for them. They must either ask their conntrymen to eat humble pie, abandon chloroform and adopt sulphuric ether as the only safe anæsthetic, or else, imitating an example which they have so often ridiculed, concentrate the labor of their committee in a grand expiring effort at "whitewashing" the terrible record and death roll of chloroform, which, it seems to us, they can never dare to display in all its naked truth.

